# The impact of physical activity on people with idiopathic pulmonary fibrosis and the associated experience – a mixed methods structured review

**DOI:** 10.1007/s11845-025-04232-8

**Published:** 2026-02-13

**Authors:** Kornelia M. Dembicka, David Murphy, Andrew O’Regan

**Affiliations:** 1 School of Medicine, University of Limerick, Limerick, Ireland; 2 Health Research Institute, University of Limerick, Limerick, Ireland; 3 Department of Physical Education and Sport Sciences, Physical Activity for Health Research Centre, University of Limerick, Limerick, Ireland

**Keywords:** Idiopathic pulmonary fibrosis, Longitudinal studies, Physical activity, Qualitative studies, Randomized control trials, Sedentary behaviour

## Abstract

**Introduction:**

The aim of this study is to review literature on the relationship between physical activity (PA) and idiopathic pulmonary fibrosis (IPF), specifically investigating the effect of PA on IPF outcomes reported in RCTs, the relationship between PA and IPF outcomes over time in longitudinal cohort studies and the lived experience of PA among people with IPF in qualitative studies.

**Methods:**

Following the PRISMA checklist, a structured search strategy was developed by two reviewers and applied to six databases up to June 2024. Grey literature was excluded. Randomised control trials, longitudinal studies and qualitative research papers were included. Screening was completed independently by two reviewers, followed by manual screening. Data extraction was completed manually using extraction tables.

**Results:**

Of 4,092 articles retrieved, fourteen were included: five randomised control trials and one follow-up study; five longitudinal and three qualitative studies. PA significantly improved QOL in four trials that analysed this outcome, with two studies reporting a *p*-value of < 0.001 and two others reporting *p*-values of < 0.04 and < 0.01. Longitudinal studies demonstrated low device-measured PA and high self-reported sedentary time correlated with poorer morbidity and mortality. Lower aerobic fitness measured using 6MWT correlated with higher mortality. Qualitative research identified progressing disease and respiratory symptoms as barriers, while social support and telehealth facilitated PA engagement.

**Conclusion:**

Short-term improvements are observed in physical capacity and QOL by incorporating PA in IPF management. Aerobic testing and PA correlate well with morbidity and mortality outcomes. Positive patient perspective on PA further supports PA prescription in IPF.

**Supplementary Information:**

The online version contains supplementary material available at 10.1007/s11845-025-04232-8.

## Introduction

IPF characterised by irreversible deterioration in lung function because of scarring from an unknown cause [38], affects adults over fifty years of age primarily [3]. The life expectancy of patients with IPF is reduced to a median of three to four years. Clinical manifestations include cough and dyspnoea [27]. As disease progresses, people with IPF find it more difficult to engage in PA and maintain physical fitness, resulting in rapid deterioration in physical function [33]. International guidelines recommend PA part of IPF management which can be delivered as part of pulmonary rehabilitation (PR) [37]. The Irish Thoracic Society advise that PA should be offered in a structured home exercise programme supporting PR [21]. The inclusion of PA in management of IPF is further supported by NICE guidelines [20] which recommend that PR, which includes exercise, should be offered and tailored as appropriate to IPF patients and held in an easily accessible space.

Many of the PR programmes patients with IPF undergo are designed for other respiratory conditions. Tailoring of PR to specific patient cohort can allow for more effective tailored exercise to combat the disease associated decline in physical fitness [55]. Furthermore, the impact of this decline in physical fitness on prognosis during the course of the disease has not been fully established [50]. Thus, conducting a review of RCTs will facilitate the development of IPF-specific PR programmes that will effectively enhance patients’ physical fitness or at minimum diminish the decline. Another important outcome that is affected by IPF is QOL. A recent review did not consider qualitative studies in their analysis [16]. High disease burden is exacerbated by the loss of physical capacity, thus it’s important to act to preserve patient independence.

*The aim of this study is to review literature pertaining to the relationship between physical activity (PA) and idiopathic pulmonary fibrosis, specifically investigating the effect of PA on IPF outcomes reported in RCTs, the relationship between PA and IPF outcomes over time reported in longitudinal cohort studies and the lived experience of PA among people with IPF reported in qualitative studies.*

## Methods

### Study design

A mixed methods structured review investigated the impact and experiences of PA on those with IPF. Mixed methods were chosen to minimise the limitations of focusing on just one form of data [36]. The definition of IPF was informed by American thoracic society guidelines [38]. Exercise and PA were defined as per WHO definition [8, 10], (Supplementary file 1) A PRISMA checklist was developed as per [29] checklist.

### Search strategy

Search strategy was developed collaboratively by two reviewers with input from a librarian. The following databases were included: PubMed, CINAHL, Cochrane, Embase, Medline, web of science. All studies up to June 2024 were included. Search terms used included “idiopathic pulmonary fibrosis”, “idiopathic interstitial lung disease”, “exercise”, “Physical Activity” “longitudinal study” “randomised control trial” and “QOL” (Supplementary file 1). Duplicate records were removed by Rayyan. Search was completed in June 2024.

### Inclusion and exclusion criteria

Exclusion and inclusion criteria were developed collaboratively by the research team (Table 1). Two reviewers (KD and DM) independently screened by title and then by full text. Disagreements were discussed by both reviewers and the research supervisor. Studies were included if agreement was reached. Full criteria are available in Supplementary file 1.

**Table 1:** Inclusion and exclusion criteria

Inclusion	Exclusion
• Original research • Published in English • Minimum participation levels (*N* > 30) • Duration interventions levels (> 8 weeks), participants with IPF as the primary population • Exercise or PA as the intervention	• Pilot studies • Studies of patients that had known causes of pulmonary fibrosis (by definition this cohort did not have idiopathic pulmonary fibrosis) • Studies on children • Passive interventions

### Data extraction

Extraction was divided equally between two reviewers. Data extraction tables were developed to gather and organise the data. Three tables were used based on the type of research, i.e., randomized control trials, longitudinal studies and qualitative studies (see Supplementary file 1). Headings used included type, year, number of participants, tests used and outcomes. Validated physical capacity measures including 6-min walking test (6MWT), step count and sedentary time were used. For qualitative studies, themes from patients and caregivers were identified and used to develop headings.

## Results

### PRISMA flow diagram

**Fig. 1:**
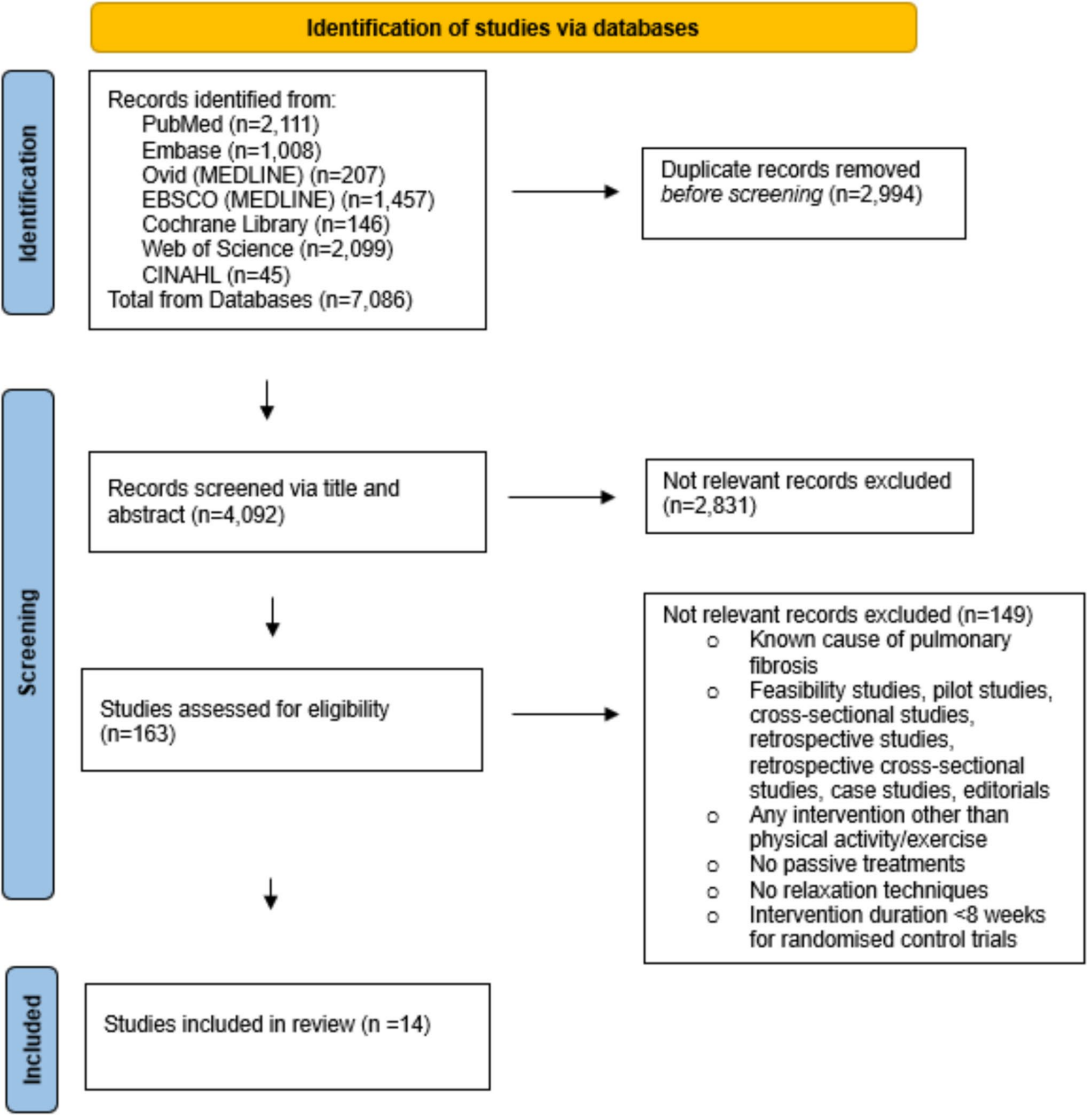
PRISMA flow diagram

The searches produced 7,086 records. Figure 1, the PRISMA flow diagram, outlines the selection process, which involved removal of duplicates and studies that did not meet the inclusion criteria. The most common reasons for exclusion were study design, study populations that did not have IPF and trials that employed interventions consisting of combinations of PA and anti-fibrotic drugs. Fourteen articles were eligible for review, including five RCTs (six studies), five qualitative and three longitudinal studies.

### Data extraction and analysis of RCTs

The five RCTs and one follow-up of a RCT study (Table 2) included in this review were conducted in Israel [48, 49], Australia [15, 19], Japan [25] and China [25]. Study population ranged from 32 [48] and 96 participants [56]. Intervention duration ranged from eight weeks (47, 46, 45] to 52 weeks [25]. All RCTs conducted the 6MWD, test for physical fitness or endurance, at baseline and post intervention period. Other tests used to measure physical fitness or physical capacity conducted include the St. George’s Respiratory Questionnaire (SGRQ-I) [25, 56], grip strength with hand-held dynamometry (HHD) [15], cycle ergometry measuring aerobic fitness [25], and functional lower extremity strength testing using 30 s sit to stand (30CST) [48, 49]. Investigation into maintenance of physical function benefits was conducted in all but one of the RCT [25]. In four out of the five RCT the PA intervention was delivered twice weekly [15, 19, 25, 48]. Zhou et al. [56] delivered the intervention twice a day, five days per week. Stationary cycling was specified as an aerobic exercise modality of choice in four of the RCTs [15, 19, 25, 56]. For resistance training, upper limb endurance training was specified in one study [19], while another RCT [15] included both upper and lower limb resistance training. Furthermore, four of the RCTs investigated the effects of PA on QOL. Measurement scales for QOL included SGRQ [15, 19, 48] and chronic respiratory disease questionnaire (CRDQ) [15, 19]. Finally, all studies had a drop-out of IPF participants, with reasons reported ranging from death as a result of their respiratory condition [56], undergoing lung transplantation [15] or simply, withdrawal of consent [48]. There were no adverse events reported in three of the RCTs [15, 19, 48]. In the study by Zhou et al. [56], 10 patients experienced adverse events, including 6 in the control group. Safety of PA interventions was further examined in the study by Kataoka et al. [25], which determined the incidence of adverse events to be equal between exercise and control groups.

**Table 2:** Overview of RCTs

Author, Year, Title, Location	Number of IPF participants	Modality of exercise included in the intervention	Intervention duration	Increase in PA/fitness/capacity on completion of intervention (Y/N)	Follow-up 6MWD findings	QOL findings	Drop-out of participant (Y/N)
Dowman, [15], The evidence of benefits of exercise training in interstitial lung disease: a randomised controlled trial, Australia	61	Aerobic exercise, cycling, walking, upper & lower limb resistance training × 2/7; home exercise prescribed	8 weeks	Y as per 6MWD	Improvement of 21 m	Y	Y: Unwell from non-respiratory related issues, exacerbation of IPF, without explanation, personal issues, withdrawal, deceased, declined follow-up, underwent transplant
Holland, [19], Short term improvement in exercise capacity and symptoms following exercise training in interstitial lung disease, Australia	34	Stationary cycling, walking training, upper limb endurance training & functional strength training × 2/7, home exercise programme once supervised programme established	8 weeks	Y as per 6MWD	Decline in 6MWD by −44.2m, not statistically significant	Y—study not powered to adequately assess this outcome	Y
Kataoka, [25], Long-term effect of pulmonary rehabilitation in idiopathic pulmonary fibrosis: a randomised controlled trial, Japan	88	Cycle ergometer, walking and resistance training using weights/body weight × 2/7 for 12 weeks, followed home rehab for 40 weeks- including calf raises, squatting and outpatient rehab minimum once every 4 weeks	52 weeks	N as per 6MWD as return to baseline (primary outcome), Y as per endurance time (secondary outcome)	N/A	N/A	Y: death, adverse events, withdrew consent
Vainshelboim, [48], Exercise Training-Based Pulmonary Rehabilitation Program Is Clinically Beneficial for Idiopathic Pulmonary Fibrosis, Israel	32	Aerobic, resistance and flexibility exercises and breathing exercises × 2/7; first 6 weeks interval training used for aerobic components, and single set system for resistance and flexibility. Next 6 weeks—aerobic endurance and multiple set system implemented	12 weeks	Y as per 6MWD	[49]	Y	Y: acute exacerbation, withdraw of consent
Vainshelboim, [49], Long-Term Effects of a 12-Week Exercise Training Program on Clinical Outcomes in Idiopathic Pulmonary Fibrosis, Israel	32	As per RCT by Vainshelboim et al. [48]	12 weeks	Follow-up of RCT at 8 months revealed no prognostic benefits and long-term maintenance	Test group: mean difference −1m; Control group: mean difference −49; not statistically significant	QOL improvement significant at 11 month follow up	Y: death, did not wish to complete study
Zhou, [56], Pulmonary Daoyin as a traditional Chinese medicine rehabilitation programme for patients with IPF: A randomized controlled trial, China	96	Group 1: Pulmonary Daoyin × 5/7 Group 2: Warm-up, stationary cycle ergometer, relaxation training × 5/7	8 weeks	Y as per 6MWD, Pulmonary Daoyin more successful than non-specific exercise programme	Difference of 6MWD for exercise and control groups was highest at 4-month follow-up	Y from baseline, No difference between the two groups i.e. pulmonary daoyin and exercise group	Y: death due to acute exacerbation of IPF, without explanation, no interest

Extraction of data from RCTs. Different modalities of PA were utilised across studies, from aerobic exercise to pulmonary Daoyin, traditional Chinese rehabilitation programme. Primary outcome of RCTs was change in physical fitness or physical capacity as per 6-min walking distance test (6MWD) and/or endurance time. Another outcome examined was Quality-of-life (QOL), measured via wellbeing scales

The first objective of this study was to analyse the effect of PA interventions on physical fitness, physical capacity and QOL. Statistically significant increase in 6MWD was seen in four of the five RCTs, achieving p-values ranging from 0.001 [48, 56], to 0.01 [19]. In the study by Kataoka et al. [25] no statistical significance was found in 6MWD with a p-value of 0.38. However, the change in endurance time was statistically significant in this study with a p-value of 0.019. Changes in HHD were not statistically significant following intervention [25]. In the 30CST test of leg strength a statistically significant difference was found with a *p*-value of < 0.001 post intervention [48]. The percentage of patients who did not improve in 6MWD was smaller for IPF groups at 30%, than for patients with connective tissue disease-related interstitial lung disease groups at 50% following intervention [15]. When comparing pulmonary Daoyin with aerobic exercise, a statistically significant difference was found with a *p*-value of < 0.044 [56]. An increase in physical fitness on completion of intervention was reported in all studies as per 6MWD, except in study by Kataoka et al. in [25]. QOL was significantly improved by PA interventions improved by PA in IPF patients, with *p*-values of < 0.001 being reported for SGRQ and < 0.04 and < 0.01 for CRDQ when comparing pre- and post-intervention results [15, 19, 48, 56]. Further details on baseline 6MWD, mean difference of 6MWD at completion and follow-up QOL findings can be found in Supplementary file 1.

### Data extraction and analysis of longitudinal studies

The second objective was to examine the relationship between PA and IPF using longitudinal studies. The three longitudinal studies included (Table 3) were conducted in Spain [1], Israel [51] and Germany [2]. The number of participants ranged from 22 to 34. The duration of follow up was one year to 34 months. Devices measuring activity were used including daily step count, sedentary time, weekly walking times and time spent physically active. Vainshelboim et al [51] included a self-reported PA questionnaire. Physical fitness testing including 6MWT, strength testing and pulmonary function testing. The hospital anxiety and depression score (HADS) and SGRQ were used as QOL measures in one of the studies [1]. Reduced activity was demonstrated overtime [2]. Studies reported that increased sedentary time was associated with increased risk of mortality and higher disease burden [1, 51]. Sitting for longer than ten hours increased mortality 21.2 hazard ratio (95% CI [4.1–32.6]; *p* trend = 0.018) and risk of hospitalisation 5.8 hazard ratio [2.2–8.4]; p trend = 0.036) [51]. Sedentary behaviour was associated with a poorer Gender-Age-Physiology (GAP) score which is a validated measure of mortality in IPF [48, [7]. Lower baseline levels of aerobic fitness and PA were associated with more severe symptoms, lower survival rates at follow up, higher rates of hospital admission and depression [51]. Muscle strength and depression were reported to be independent predictors of mortality [1].

**Table 3:** Data extraction of longitudinal studies

Author Year Title Location	Participant demographics N = NUMBER L = LOST TO FOLLOW UP	PA measure/Sedentary measure	QOL measure	Physical functional evaluation	Pulmonary function test	Physical functional trend over time	Prognostic correlation
Vainshelboim [51] Lifestyle Behaviours and Clinical Outcomes in Idiopathic Pulmonary Fibrosis Israel	N 34 Male 22 Female 12 Age 50–81 L11	Weekly walking times Daily sitting times Sedentary time	N	PA questionnaire 6MWT Cardiopulmonary testing	FVC DLCO	Decreased 6MWT distance Decreased walking times Increased sedentary time	Increased mortality and morbidity with longer sitting time Increased mortality with less walking time Increased mortality and morbidity with lower FVC AND DLCO
Badenes Bonet Predictors and changes of PA in idiopathic pulmonary fibrosis 2022 Spain	N 22 Male 30 Female 10 L 8 Age 64–79)	Daily step count Daily minutes of moderate-to-vigorous PA Sedentary time	HADS SGRQ	6MWT Quadricep strength	FVC DLCO	Decreased 6MWT SPO2 Increased sedentary time	Increased mortality with lower SPO2 scores on 6MWT Lower DSC increased risk of mortality Increased mortality with lower FVC AND DLCO
Bahmer Prognosis and longitudinal changes of PA in idiopathic pulmonary fibrosis 2017 Germany	N 46 Male 32 Female 12 L 20 Age 61–84	Daily step count	N	6MWT	FVC DLCO	Decreased 6MWT distance Decreased DSC Decrease PA up to 50%	Lower DSC increased mortality risk Decreased walking distance increased mortality risk Increased mortality with lower FVC AND DLCO

Extraction of data from three longitudinal studies. *DSC* Daily step count measured with wearable activity tracker; *6MWT* 6 min walk test; *FVC* Forced vital capacity measured by spirometry; *HADS* Hospital anxiety and depression score (wellbeing score) SGRQ: St George Respiratory Questionnaire (wellbeing score) DLCO:Diffusing capacity for carbon monoxide. FVC:Forced vital capacity

Longitudinal study results report a relationship between lower PA/fitness scores and increased mortality and morbidity. Various measures of fitness were used all showing a similar trend. Simple measures used such as 6MWT, DSC, quadricep strength and questionnaires provided valuable insight.

### Data extraction and analysis of qualitative studies

Our third objective was to explore IPF sufferers experience with PA and identify barriers and facilitators to participation. Table 4 summarises the five qualitative studies included in this review. Studies were located in the UK [41], Australia [9], India [18], Sweden [24] and Ireland [33]. Sample size ranged from ten to one hundred. Two studies [18, 33] focussed on patient experiences with home-based pulmonary rehab. Main themes included (a)perceived effects of PA, (b)facilitators and barriers to PA and (c)suggestions for facilitating PA.

**Table 4:** Data extraction from qualitative studies

Author Year Title Location	Population No. of participants Age Gender	Facilitators barriers	Advantages disadvantages *	Suggestions for increased participation
Burnett [9] Understanding the patient’s experience of care in idiopathic pulmonary fibrosis’ Australia	N 100 Median Age 69.5 year 17 Male 3 Female Caregiver: 5	social support family support education, fear, environment, unable to complete former hobbies	increased sense of wellbeing, social, improved sleep, difficult to complete, low energy	encouragement, education, involving family members, group exercise
O’Shea [33] A qualitative exploration of people living with idiopathic pulmonary fibrosis experience of a virtual pulmonary rehabilitation program Ireland	N 13 Age average 69.5 7 Male 6 Female	social support, home based/online, feeling safe comparing to others, technology, comorbidities, functional ability	improved sense of wellbeing, renewed hope, less symptomatic fun, enjoyable no change to symptoms, disease progression	participation in virtual pulmonary rehab, more frequent classes, appropriate times
Jernås [24] Experiences of living with idiopathic pulmonary fibrosis in relation to PA—“how the hills became steeper and steeper”: a qualitative interview study, Sweden	N 14 Age average 77 10 Male 4 Female	support from HCP, social support, adapted exercise, appropriate planning symptoms	improved sense of wellbeing, symptom relief, slowed disease progression, feeling of reward reduced PA, fatigue, pain	create coping strategies, support systems, adapted exercise based on phases of disease
Hanif [18] Understanding the lived experience of idiopathic pulmonary fibrosis and how this shapes views on home-based pulmonary rehabilitation in Delhi, India	IPF: N 20 Median age 69.5 year 17 male 3 female Caregiver: N 20	contact with HCP, social support, adapted activity, planning symptoms, travel, financial burdens, disability	increased independence, improved symptoms, reduced perception of hospital admission, slow disease progression O2 desaturation, lack of interaction with home-based rehab, symptoms	education, leaflets, involve families, increased access to HCP

Extraction of data from five qualitative studies included in this review. *HCP* Healthcare professional; *IPF* Idiopathic pulmonary fibrosis

**Perceived effects of PA** included wellbeing, activities of daily living, increased independence, positive social interaction and reduced physical symptoms [9, 18, 33]. Disadvantages included increased symptoms in some patients, the need to modify activities as disease progressed, the inability to complete previous PA or hobbies and seeing no benefit [9, 18, 24, 33, 41]. Caregivers noted similar experiences to participants [18]. QOL was reported as poorer when PA tolerance was low [18, 24]. Facilitators included contact with health care professionals, social support, suitable times, online classes and health care professionals educating patients for self-management [9, 18, 24, 33, 41]**. Suggestions for facilitating PA** included increased contact with healthcare professionals (HCPs), education, involving family/caregivers and involving support groups [9, 18, 24, 33, 41].

## Discussion

The aim of this study was to review the literature pertaining to the relationship between PA and idiopathic pulmonary fibrosis. Wide ranging PA interventions in RCTs demonstrated positive effects on physical fitness or physical capacity and QOL of patients with IPF, with statistically significant differences seen in these outcomes. Longitudinal studies reported that increase in sedentary time and lower levels of PA accompanying IPF disease progression was associated with a poorer GAP score, i.e. higher mortality in IPF. Qualitative papers outlined how perceived wellbeing is improved by being physically active and that enhancing the social impact of PA interventions is a key facilitator for PA engagement.

All of the RCTs included in this review reported improved 6MWD or endurance time on completion of interventions, regardless of the intensity of the PA intervention [15, 19, 25, 48, 56]. Therefore, engaging people with IPF in the most feasible form of PA is recommended. This finding is consistent with results from a previous systematic review by Hanada et al. 17, who looked at exercise training for patients with IPF in combination with breathing exercises. Similarly, patients with chronic obstructive pulmonary disease (COPD), bronchiectasis and cystic fibrosis also demonstrate improvement in exercise capacity after incorporating various forms of PA interventions, both supervised and unsupervised [13, 34, 53]. Furthermore, the four RCTs that studied QOL as an outcome reported improvement in wellbeing measurement scales. In another review, which included 190 participants, it was found that pulmonary rehabilitation may enhance PA capacity and improve QOL [54]. However, physical fitness or physical capacity benefits from PA interventions are not always maintained at follow up with variability between the 6MWD reports in the months post intervention [15, 19, 25, 48, 56]. The benefits observed during PA interventions are not maintained unless a post-rehabilitation/intervention programme is introduced. A study on COPD patients, reported that participants were able to sustain improved exercise capacity following an 8-weeks PR with maintenance programmes that were either supervised outpatient programmes or with unsupervised, home-based programmes. It was hypothesised that one of the reasons for maintenance of exercise capacity in the unsupervised and home-based group was the ongoing testing and follow-up [44]. Such approach to maintenance programmes should be investigated in future PA intervention studies for IPF patients. The 11-month follow-up study by Vainshelboim, [49], reported some maintenance in the improvements achieved through the trial in QOL. This is supported by another RCT, where QOL was deemed the outcome with best medium-term maintenance [23]. Finally, this review demonstrated trials including patients with IPF were regularly affected by dropping out of participants [15, 19, 25, 48, 56]. Non-completion of trials by IPF patients is a commonly reported limitation of studies and patients further along the disease course are more likely to drop-out due to disease deterioration [22, 31, 43].

The relationship between reduced physical capacity and PA levels and morbidity and mortality outcomes are established [28, 46]). Reduced PA, physical function and lung function scores occurred over time as disease progressed. Our longitudinal results confirm this in an IPF context. Aerobic fitness is arguably the strongest predictor of outcome in the general population [46], which can be measured simply by the 6-min walk test [39, 45]. The 6MWT is also an effective predictor of mortality for COPD and bronchiectasis [5, 14]. Our finding of lower leg strength measured by 30 CST strength correlating with morbidity is repeatable in other non-communicable diseases such as lung cancer, diabetes and cardiovascular disease [30]. This indicates that some form of physical fitness testing may provide insight as measure of disease progression or an indicator of mortality in this cohort, but further studies are indicated given the sample size.

The final novel contribution of this review is the positive experiences reported by this cohort when partaking in group PA interventions. This is a strong theme throughout our results, which is also seen in other chronic conditions [11, 42, 47] and recommended to older adults by the WHO [35]. Existing research shows group PA can provide a wider benefit than just physical, which is another added benefit [32]. Group activity can be seen as positive from a healthcare point of view, as delivering interventions such as PR is less resource intensive Rubi et al. [40]. Our results show an online platform to deliver group PA is an acceptable alternative [33] which concurs with existing literature [12], Bennet et al. [4]. This can ease environmental and access barriers to participation [33]. Providing group intervention based on our results may help common barriers such as social support, safety concerns and education when delivered by a HCP. Engagement in PA long-term is a worthwhile goal but remains a challenge. Completing group PA can help with social connection which may keep patients involved longer [32].

The key strength of this study was the use of a combination of three types of methodologies which gives a fuller perspective of the benefits, long term outcomes and challenges associated with this disease in relation to PA. Our novel review of qualitative research in relation to IPF and PA collates data from several sources which allows easy access of information to HCPs, policy makers and researchers. Main limitation of the study is that research published in languages other than English may have been missed. Additionally, the limited number of RCTs included due to strict inclusion criteria and lack of meta-analysis did not permit determination of optimal modality of exercise for IPF patients. Nonetheless, benefits of engaging in structured programmes are described and provide direction for incorporation of PA as a modality of management. Critical appraisal tools were not used to appraise research quality as this was beyond the scope of this study as outlined in protocol.

Pulmonary rehabilitation services in Ireland have previously been studied for their care of patients with IPF. It was revealed that all surveyed sites were tailoring the exercise component of rehabilitation to patients’ need or ability, yet only four out of eighteen sites reported providing IPF-specific education [52]. This reiterates what was recognized in this study, that a lot of resources are deployed to optimise short inpatient or outpatient supervised PA programmes, or even more recently virtual rehabilitation programmes [33]. However, not enough is being committed to establishing maintenance programmes. Further research is needed to form accessible and feasible home-based or community-based maintenance programmes that can continue over different phases of disease. Future research should also focus on modalities of PA that can provide long-term effects and can be modified as needed, as maintenance of some benefits on physical fitness, physical capacity and QOL remains an issue. It has been suggested by [50], to utilise electronic systems in future studies, as these are objective and accurate and may help overcome any ambiguity on duration of impact on PA in patients with IPF. Furthermore, future research must recognize that there is a global deficit to-date in health services in delivering early IPF disease diagnosis, which inevitably affects timely access to disease therapy and management, including PR [6]. Thus, investigation into practice from point of initial presentation to final diagnosis should be considered. This would facilitate early intervention of PA as a modality of management, leading to better health outcomes. Finally, increasing exercise capacity in pulmonary diseases can be done not only by incorporating exercise training into pulmonary rehabilitation, but also by behavioural interventions and web-based interventions [26]. While these interventions are already receiving attention, focused research into each individual intervention needs to be combined with comparison between the different approaches in future research. Continued research should be completed in the delivery of online activities especially in the context of a growing online world and a population that will be more technology literate. Continued research in the context of IPF is needed to confirm the use of physical testing as a predictor disease progression.

## Conclusion

PA is associated with improvements in physical fitness and QOL, but further research is needed to demonstrate the sustainability of these benefits. Longitudinal studies demonstrated an association between PA levels and morbidity/mortality. Evidence from qualitative research supports social support as a mediator to sustain PA behaviour in this cohort.

## Supplementary Information

Below is the link to the electronic supplementary material.

Supplementary File 1 (DOCX 36.4 KB)

## Data Availability

The authors confirm that the data supporting the findings of this study are available within the article and/or its supplementary materials.

## Abbreviations

30CST: 30 Second chair stand test
6MWD: 6-Minute walking distance
6MWT: 6-Minute walking test
CRDQ: Chronic respiratory disease questionnaire
GAP: Gender-age-physiology index
HADS: Hospital anxiety and depression score
HCP: Healthcare professional
HHD: Hand-held dynamometry
IPF: Idiopathic pulmonary fibrosis
PA: Physical activity
PR: Pulmonary rehabilitation
QOL: Quality of life
RCT: Randomised control trial
SGRQ: St George’s Respiratory Questionnaire
WHO: World health organization
