# Supplementary material for: The impact of physical activity on people with idiopathic pulmonary fibrosis and the associated experience – a mixed methods structured review
Source: Ir J Med Sci. 2026 Feb 13;195(3):1413–22. doi: 10.1007/s11845-025-04232-8 (PMC13342121; doi:10.1007/s11845-025-04232-8)
Supplement: Supplementary file 1 — (DOCX 36.4 KB) [file 11845_2025_4232_MOESM1_ESM.docx]

**Appendices:**

***Appendix 1: Study Protocol***

***Appendix 2: Index of studies reviewed***

**Appendix 1.**

Structured Review Protocol

**1.      Population of Interest, Intervention, Comparator, Outcome**

| **Population of interest:** | Patients with idiopathic pulmonary fibrosis regardless of age gender ethnicity, comorbidities |
| --- | --- |
| **Intervention:** | Exercise, PA, physical training, supervised exercise, aerobic training, resistance training |
| **Comparator:** | Not applicable, no exercises, typical treatment |
| **Outcome:** | Patient experiences with PA, trial data exercise vs no exercise, relationship with PA overtime. |

2.      **Search**

**Topic a** = idiopathic pulmonary fibrosis OR Familial Idiopathic Pulmonary Fibrosis OR idiopathic interstitial lung disease OR cryptogenic fibrosing alveolitis OR fibrosing alveolitis OR lung fibrosis OR interstitial pneumonitis

AND

**Topic b** = exercise OR PA OR exertion OR aerobic training OR aerobic exercise OR exercise training OR isometric exercise OR physical exercise OR resistance training OR resistance exercise OR weight training OR physical training OR dance OR pulmonary rehab OR pulmonary rehabilitation OR supervised exercise OR group exercise OR group training OR walking OR cycling OR home rehabilitation OR full body exercise

AND

**Topic c** = longitudinal study OR longitudinal outcomes OR randomised control trial OR RCT OR experiences OR Life Quality OR Health-Related QOL OR Health Related OR QOL OR HRQOL

**Documenting the search –**

 1. Database searched - Embase, EBSCO, Web of science, OVID (Medline), Cinahl, Pubmed, Cochrane

2. Time coverage: no start date to 06/06/24

3. Search strategies for each database: Boolean search used as per individual databases, assistance from university librarian with searches.

4. The date the search was conducted: 06/06/24

5. The number of results from the search: 7086

6. The number of results after deduplication: Total studies uploaded to Endnote: 7086; Deduplication on endnote: 4969; Deduplication on Rayyan: 4092

**3.**      **Screening**

**Criteria:**

| **Inclusion** | **Exclusion** |
| --- | --- |
| Patients with Idiopathic Pulmonary Fibrosis (IPF) | Known causes of pulmonary fibrosis |
| RCTs, longitudinal cohort studies, qualitative studies | Feasibility studies, pilot studies, cross-sectional studies, retrospective cross-sectional studies, case studies, editorial |
| PA or fitness recorded as study outcome for RCTs;  Longitudinal studies relating QOL with PA/exercise;  Qualitative studies of perceptions/experiences with PA of patients or caregivers | Any interventions other than PA/exercise |
| All types of PA and/or exercise as the primary metric or intervention | No passive treatments; no airway clearance interventions |
| Studies based on adults only | No relaxation techniques such as mindfulness |
| Thirty participants or more | Intervention duration less than 8 weeks for RCTs |
| English language |  |

Screening performed by KD and DM

        i.            Title and abstract

   ii.            Full paper

**4.**      **Analysis**

Review study description; population description; intervention description; monitoring description

What form of PA was used as the intervention? What was the duration of the intervention? Exercise tests conducted? How long were patients followed up, and what was the outcome during the follow-up? Was there an increase in PA/fitness/capacity on completion? Drop-out of participants?

In qualitative studies, what was the data collection method? What were the benefits/advantages discussed? What were the barriers that were discussed? What was the outcome/conclusion of the qualitative study?

In longitudinal studies what was the quality-of-life questionnaire used? What was the Quality-of-life outcome? Was there a fitness outcome and how was it measured/documented?

**5. Definition of physical activity and exercise**

Physical activity is defined as any bodily movement produced by skeletal muscles that results in energy expenditure.

Exercise is a subset of physical activity that is planned, structured, and repetitive and has as a final or an intermediate objective the improvement or maintenance of physical fitness

As per:

Bull, F.C. *et al.* (2020) ‘World Health Organization 2020 guidelines on PA and sedentary behaviour’, *British Journal of Sports Medicine*, 54(24), pp. 1451–1462. doi:10.1136/bjsports-2020-102955.

Caspersen CJ, Powell KE, Christenson GM. Physical activity, exercise, and physical fitness: definitions and distinctions for health-related research. Public Health Rep. 1985 Mar-Apr;100(2):126-31. PMID: 3920711; PMCID: PMC1424733

**5.      Tables**

Table 1: RCTs Data Extraction

| **Study title** | **Publication Year** | **Country** | **Study type** | **Number of IPF participants** | **Method of intervention supervised/unsupervised i.e. inpatient/clinic or home** | **Modality of exercise included in the intervention** | **Duration of intervention** | **Exercise tests conducted** |
| --- | --- | --- | --- | --- | --- | --- | --- | --- |
| Pulmonary Daoyin as a traditional Chinese medicine rehabilitation programme for patients with IPF: a randomized controlled trial | 2021 | China | RCT | 96 | Supervised and unsupervised | Group 1: Pulmonary Daoyin x 5/7 Group 2: Warm-up, stationary cycle ergometer, relaxation training x 5/7 | 8 weeks | 6MWD, SGRQ-1 |
| The evidence of benefits of exercise training in interstitial lung disease: a randomised controlled trial | 2017 | Australia | RCT | 61 | Supervised and unsupervised | Aerobic exercise, cycling, walking, upper & lower limb resistance training x 2/7; home exercise prescribed | 8 weeks | 6MWD, hand-held dynamometry (knee extensor, elbow flexor strength) |
| Short term improvement in exercise capacity and symptoms following exercise training in interstitial lung disease | 2008 | Australia | RCT | 34 | Supervised and unsupervised | Stationary cycling, walking training, upper limb endurance training & functional strength training x 2/7, with a home exercise programme once supervised programme established | 8 weeks | 6MWD |
| Long-term effect of pulmonary rehabilitation in idiopathic pulmonary fibrosis: a randomised controlled trial | 2023 | Japan | RCT | 88 | Supervised and unsupervised | Cycle ergometer, walking and resistance training using weights/body weight x 2/7 for 12 weeks, followed by at home rehab for 40 weeks including calf raises & squatting and outpatient pulmonary rehab minimum once every 4 weeks | 52 weeks | 6MWD, daily steps, SGRQ-1, cycle ergometry |
| Long-Term Effects of a 12-Week Exercise Training Program on Clinical Outcomes in Idiopathic Pulmonary Fibrosis | 2015 | Israel | RCT | 32 | Supervised | Aerobic, resistance and flexibility exercises and breathing exercises twice weekly, with first 6 weeks interval training being used for aerobic components, and single set system for resistance and flexibility. Next 6 weeks - aerobic endurance and multiple set system implemented. | 12 weeks | Cardiopulmonary exercise test (CPET), 6MWD, 30-S chair stand |
| Exercise training-based pulmonary rehabilitation program is clinically beneficial for idiopathic pulmonary fibrosis | 2014 | Israel | RCT | 32 | Supervised | Aerobic, resistance and flexibility exercises and breathing exercises twice weekly, , with first 6 weeks interval training being used for aerobic components, and single set system for resistance and flexibility. Next 6 weeks - aerobic endurance and multiple set system implemented. | 12 weeks | Cardiopulmonary exercise test (CPET), 6MWD, 30-S chair stand |

Continuation of Table 1:

| **Study title** | **Time from completion to follow-up** | **Increase in PA/fitness/capacity on completion of intervention (Y/N)** | **Mean 6MWD (m) at baseline** | **Mean difference 6MWD (m) at completion of intervention** | **Follow-up 6MWD findings** | **Drop-out of participants (Y/N)** | **QOL measurement tools** | **Improvement in QOL on completion of intervention (Y/N)** | **Follow-up QOL findings** |
| --- | --- | --- | --- | --- | --- | --- | --- | --- | --- |
| Pulmonary Daoyin as a traditional Chinese medicine rehabilitation programme for patients with IPF: a randomized controlled trial | 4 months | Y as per 6MWD, Pulmonary Daoyin > non-specific exercise programme | Group 1: 284.06; Group 2: 279.10 | Group 1: improvement by 60.44; Group 2: improvement by 32.16 | Difference for exercise and control groups was highest at 4 month follow-up | Y: death due to acute exacerbation of IPF, without explanation, no interest | SGRQ‐I | Y from baseline, No difference between the two groups i.e. pulmonary daoyin and exercise group | Increase in SGRQ-I scores at follow-up in both intervention groups |
| The evidence of benefits of exercise training in interstitial lung disease: a randomised controlled trial | 4 months | Y as per 6MWD | 430 | Improvement by 25 | Improvement of 21 m | Y: Unwell from non-respiratory related issues, exacerbation of IPF, without explanation, personal issues, withdrawal, deceased, declined follow-up, underwent transplant | SGRQ-1 and CRDQ | Y as per SGRQ-1 and CRDQ | Decline in HRQOL benefits at follow-up |
| Short term improvement in exercise capacity and symptoms following exercise training in interstitial lung disease | 4 months | Y as per 6MWD | 354 | Improvement by 25.1 | Decline in 6MWD by -44.2m, not statistically significant | Y | Medical Outcomes Study Short Form 36 (SF-36), CRDQ | Y - study not powered to adequately assess this outcome | Decline in Quality-of-life measures at follow up |
| Long-term effect of pulmonary rehabilitation in idiopathic pulmonary fibrosis: a randomised controlled trial | No follow-up | N as per 6MWD, Y as per endurance time | Pulmonary rehab group: 447; Control group: 459 | Pulmonary rehab group: Reduced by 33; Control group: reduced by 53. Not statistically significant; return to baseline. Endurance time increased in pulmonary rehab group from 372 s to 441 af 52 weeks; in control group endurance time decreased from 396s to 275s. | N/A | Y: death, adverse events, withdrew consent | Nil | N/A | N/A |
| Long-Term Effects of a 12-Week Exercise Training Program on Clinical Outcomes in Idiopathic Pulmonary Fibrosis | 8 months | N/A - on completion data analysed in a different study | Test group: 479, Control group: 526 | N/A - on completion data analysed in a different study | Test group: mean difference -1m; Control group: mean difference -49; not statisticall significant | Y: death, did not wish to complete study | SGRQ-I | (Vainshelboim et al, 2014) | QOL improvement significant at 11 month follow up |
| Exercise training-based pulmonary rehabilitation program is clinically beneficial for idiopathic pulmonary fibrosis | Follow-up part of different study | Y as per 6MWD | Test group: 471; Control: 513 | Test group: improvement by 70.4; Control group: reduction in 6MWD by -10.6; mean difference: 81 statistically significant | Follow-up part of different study | Y: acute exacerbation, withdraw of consent | SGRQ-I | Y as per SGRQ-I | (Vainshelboim et al, 2015) |

Table 2: Data extraction Longitudinal Studies

| **Study title** | **Publication Year** | **Country** | **Number of participants IPF** | **Duration** | **PA monitored** | **PA measure** | **QOL outcome** |
| --- | --- | --- | --- | --- | --- | --- | --- |
| Lifestyle Behaviours and Clinical Outcomes in Idiopathic Pulmonary Fibrosis | 2018 | Israel | 34 | 1 YEAR | weekly walking times, daily sitting times, walkig, sedentary time | International Physical Activity Questionnaires (IPAQ), body fat, cardiopulmonary testing, 6MWD, walking time | Nil |
| Predictors and changes of physical activity in idiopathic pulmonary fibrosis | 2022 | Spain | 22 | 1 YEAR | daily step count, daily minutes of moderate-to-vigorous PA, sedentary time | accelereometer,6MWT, body composition, muscle strength, pulmonary function tests, modified research council (mrc) dyspnoea scale | HADS |
| Prognosis and longitudinal changes of physical activity in idiopathic pulmonary fibrosis | 2017 | GERMANY | 46 | 34MONTHS | daily step count | FVC, diffusing capacity of the lungs for carbon monoxide (DLCO) and 6MWD. | Nil |

Continuation of Table 2:

| **Study title** | **Fitness outcome** | **Fitness trend** | **Prognostic correlation sedentary** | **Prognostic correlation activity** | **Lost to follow up** |
| --- | --- | --- | --- | --- | --- |
| Lifestyle Behaviours and Clinical Outcomes in Idiopathic Pulmonary Fibrosis | 6MWD decline correlated with sedentary time and weekly walking times | N/A | increased risk of hospitalisation death with>5 hours sitting time | Increased risk of death/hospitalisation <100 mins walking | 11 died  Age, years 68 [50 – 81] Male/Female 22 (65)/12 (35) |
| Predictors and changes of physical activity in idiopathic pulmonary fibrosis | Strength preserved | lower step correlated with poor 6MWT, strength score, lower depression score. | Sedentary behaviour associated with presence of GAP III stage, i.e. lowest stage correlating with highest risk mortality | Lower step count, Lower 6MWT, poorer lung function testing, dlco, correlating with morbidity mortality, muscle strength and depression independent predictors of mortality | 11 lost follow up, death, lung transplant  71.4 (6.5) years, and 30 men 10 female were male |
| Prognosis and longitudinal changes of physical activity in idiopathic pulmonary fibrosis | N/A | PA decrease up to 50% | Increased risk of mortality as per GAP stage/score, lower 6MWT, pulmonary function, Increased risk of larger decline IN PA VS 6MWT  Dlco, fvc correlated with increased risk of death | Increased risk of death - non survivors had lower exercise capacity and PA at baseline compared to survivors. PA predicts mortality of IPF patients, however, longitudinal decline in PA seems to be disproportionally large | 20 lost to follow up death  46 IPF-patients (mean age, 67 years  34 men 12 women |

Table 3: Data Extraction Qualitative Studies

| **Study title** | **Publication Year** | **Country** | **Study type** | **Number of participants IPF** | **Number of participants caregivers /HCP** | **Patient advantages** | **Patient disadvantages** | **Caregiver/ HCP advantages** | **Caregiver/ HCP disadvantages** |
| --- | --- | --- | --- | --- | --- | --- | --- | --- | --- |
| Understanding the lived experience of idiopathic pulmonary fibrosis and how this shapes views on home-based pulmonary rehabilitation in Delhi, India | 2024  median age is 69.5 year  17 MALES 3 FEMALES | India | Qualitative study using semi-structured interviews | 20 | 20 | Health benefit not specified, possibility of improving condition and ability to carry out activities of daily living, increased independence with home-based pulmonary rehab | Dyspnoea limiting participation, reduced QOL with reduced exercise tolerance | improved symptoms, dyspnoea, reduced hospital admission, slow level of decline, home based pulmonary rehab would help overcome barriers like travel, disability, geographical restrictions and financial burdens | O2 desaturation, lack of interaction with home-based rehab, |
| Experiences of living with idiopathic pulmonary fibrosis in relation to physical activity - “How the hills became steeper and steeper”: a qualitative interview study | 2024  77 years old  4 FEMALES  10 MALES | Sweden | Qualitative study using one-to-one semi-structured interviews | 14 | 0 | increased sense of wellbeing, physiotherapy perceived as positive part of healthcare treatment giving motivation to maintain PA and strengthening physical capacity leading to symptom relief and slowed down disease progression, feeling of reward after doing well | Reduced PA limiting QOL, fatigue limiting pa, need to plan PA e.g. avoiding hilly terrains, walking too long. | N/A | N/A |
| A qualitative exploration of people living with idiopathic pulmonary fibrosis experience of a virtual pulmonary rehabilitation programme | 2022  13 participants  Age 69.5(10.4) years; 7M:6F | IRELAND | Qualitative study using one-to-one semi- structured interviews | 13 | 0 | Improved sense of wellbeing, improved mental wellbeing, renewed hope, less symptomatic with cough and SOB, fun, enjoyable | no change, disease progression | more involved in care | N/A |
| Understanding the patient's experience of care in idiopathic pulmonary fibrosis | 2019  61 males  39 FEMALES  AGE  57 – 90 AGE | Australia | Qualitative study using one-to-one semi- structured interviews | 100 | 5 | improved symptoms, improved psychological wellbeing , social , slow progression of disease | Not specified | Not specified | Exercise instructors had little experience with IPF |
| Patients' experiences of coping with Idiopathic Pulmonary Fibrosis and their recommendations for its clinical management | 2018  53-81 years  9 male 1 FEMALE | UK | Qualitative study using semi- structured interviews with two of the authors | 10 | 0 | Opportunity to socialize, improved sense of mental wellbeing, renewed optimism, increased commitment to self-care, improved sleep, improved flexibility | difficulty with day-to-day activities, struggle, can't complete hobbies golf swimming, low energy | Not specified | Not specified |
| Understanding the lived experience of idiopathic pulmonary fibrosis and how this shapes views on home-based pulmonary rehabilitation in Delhi, India | 2024 | India | Qualitative study using semi-structured interviews | 20 | 20 | Health benefit not specified, possibility of improving condition and ability to carry out activities of daily living, increased independence with home-based pulmonary rehab | Dyspnoea limiting participation, reduced QOL with reduced exercise tolerance | improved symptoms, dyspnoea, reduced hospital admission, slow level of decline, home based pulmonary rehab would help overcome barriers like travel, disability, geographical restrictions and financial burdens | O2 desaturation, lack of interaction with home-based rehab, |

Continuation of Table 3:

| **Study title** | **Outcome/ Conclusion** | **Trends** | **Intervention** | **IPF impact on daily life** | **Barriers to exercise** | **Facilitators to exercise** | **Suggestions for PA** | **Number of interviews** |
| --- | --- | --- | --- | --- | --- | --- | --- | --- |
| Understanding the lived experience of idiopathic pulmonary fibrosis and how this shapes views on home-based pulmonary rehabilitation in Delhi, India | positive impact of home-based rehab | health impact, disease management, perception | home based pulmonary rehab | Breathlessness, reduced exercise tolerance | weather, family member dependence, comorbidities, guidance and supervision | home based | In depth education, mixed mode, involving caregivers ,information booklets, access to health care professional | 40 |
| Experiences of living with idiopathic pulmonary fibrosis in relation to PA - “How the hills became steeper and steeper”: a qualitative interview study | Facilitate PA, identifying barriers, different needs in different phases of disease | fear, acceptance, change in self-image | NIL | increased need for rest, change in perception of self-image, coping by keeping busy | increased shortness of breath, dry cough | contact with a HCP/physio, social support adapted activity, planning activity appropriately | Facilitate opportunities to maintain an identity of being active individuals, create coping strategies around PA and daily life, support systems that meet the different demands around different phases of the disease should be provided by the healthcare system | 14 |
| A qualitative exploration of people living with idiopathic pulmonary fibrosis experience of a virtual pulmonary rehabilitation programme | VPR is an enjoyable experience for people, VPR allowed for enhanced accessibility | The impact of VPR on health and outlook, the reality of VPR, being active after VPR and Living with IPF during the Covid-19 Pandemic | Virtual pulmonary rehab | Reduced physical fitness affecting ability to participate in social activities, severe dyspnoea and cough causing impacting will to live | Technology, comparing to others, comorbidities including pain, weather, time, reduced functional status | acceptable times, home based, social support, no commute, feeling safe, technology | Participation in virtual pulmonary rehab programmes recommended by IPF patients for other patients with same disease, suggest the programmes should be longer and more frequent, not conducted during the summer as clashed with holiday plans | 12 |
| Understanding the patient's experience of care in idiopathic pulmonary fibrosis | Clinicians should be more positive about the active role patients can take in self-care which includes exercise | nil identified | Some patients underwent pulmonary rehab | Impact on life not specified? | travel, heavy oxygen tank, difficulty with equipment, | social support, HCP guided | Clinicians should be more positive about the active role patients can take in self-care which includes exercise | 5 |
| Patients' experiences of coping with Idiopathic Pulmonary Fibrosis and their recommendations for its clinical management | provide patients with information that focuses on living with IPF, encouraging them to make lifestyle changes and adaptations to improve QOL. Family members should receive education about IPF so that they can support such changes. Patients should be encouraged to join a support group and to participate in PA (again preferably group-based) | nil identified | Unspecified exercise programmes | Isolated, stigmatised, depressed, huge impact on lifestyle with limited ability to maintain physical hobbies, need to plan all activities and reorganize living spaces, relationships affected | lack of education, fear, environment | social support family support | provide patients with information that focuses on living with IPF, encouraging them to make lifestyle changes and adaptations to improve QOL. Family members should receive education about IPF so that they can support such changes. Patients should be encouraged to join a support group and to participate in PA (again preferably group-based) | 10 |
| Understanding the lived experience of idiopathic pulmonary fibrosis and how this shapes views on home-based pulmonary rehabilitation in Delhi, India | positive impact of home-based rehab | health impact, disease management, perception | home based pulmonary rehab | Breathlessness, reduced exercise tolerance | weather, family member dependence, comorbidities, guidance and supervision | home based | In depth education, mixed mode, involving care givers, information booklets, access to health care professionals | 40 |

**Appendix 2.**

***RCTs Study References:***

1. **Dowman, L.M. *et al.* (2017)** ‘The evidence of benefits of exercise training in interstitial lung disease: A randomised controlled trial’, *Thorax*, 72(7), pp. 610–619. doi:10.1136/thoraxjnl-2016-208638.
2. **Holland, A.E. *et al.* (2008)** ‘Short term improvement in exercise capacity and symptoms following exercise training in interstitial lung disease’, *Thorax*, 63(6), pp. 549–554. doi:10.1136/thx.2007.088070.
3. **Kataoka, K. *et al.* (2023)** ‘Long-term effect of pulmonary rehabilitation in idiopathic pulmonary fibrosis: A randomised controlled trial’, *Thorax*, 78(8), pp. 784–791. doi:10.1136/thorax-2022-219792.
4. **Vainshelboim, B. *et al.* (2014)** ‘Exercise training-based pulmonary rehabilitation program is clinically beneficial for idiopathic pulmonary fibrosis’, *Respiration*, 88(5), pp. 378–388. doi:10.1159/000367899.
5. **Vainshelboim, B. *et al.* (2015)** ‘Long-term effects of a 12-week exercise training program on clinical outcomes in idiopathic pulmonary fibrosis’, *Lung*, 193(3), pp. 345–354. doi:10.1007/s00408-015-9703-0.
6. **Zhou, M. *et al.* (2020)** ‘Pulmonary Daoyin as a traditional Chinese medicine rehabilitation programme for patients with ipf: A randomized controlled trial’, *Respirology*, 26(4), pp. 360–369. doi:10.1111/resp.13972.

***Qualitative study references:***

1. **Burnett, K., Glaspole, I. and Holland, A.E. (2018)** ‘Understanding the patient’s experience of care in idiopathic pulmonary fibrosis’, Respirology, 24(3), pp. 270–277. doi:10.1111/resp.13414.
2. **Hanif, H. et al. (2024)** ‘Understanding the lived experience of idiopathic pulmonary fibrosis and how this shapes views on home-based pulmonary rehabilitation in Delhi, India’, Chronic Respiratory Disease, 21. doi:10.1177/14799731241258216.
3. **Jernås, A. et al. (2024)** ‘Experiences of living with idiopathic pulmonary fibrosis in relation to physical activity- “how the hills became steeper and steeper”: A qualitative interview study’, BMC Pulmonary Medicine, 24(1). doi:10.1186/s12890-024-03064-z.
4. **O’Shea, O. et al. (2022)** ‘A qualitative exploration of people living with idiopathic pulmonary fibrosis experience of a Virtual Pulmonary Rehabilitation Programme’, BMC Pulmonary Medicine, 22(1). doi:10.1186/s12890-022-02221-6.
5. **Senanayake, S. et al. (2018)** ‘Patients’ experiences of coping with idiopathic pulmonary fibrosis and their recommendations for its clinical management’, PLOS ONE, 13(5). doi:10.1371/journal.pone.0197660.

***Longitudinal studies  references:***

1. **Badenes-Bonet, D. et al. (2022)** ‘Predictors and changes of physical activity in idiopathic pulmonary fibrosis’, BMC Pulmonary Medicine, 22(1). doi:10.1186/s12890-022-02134-4.
2. **Bahmer, T. et al. (2017)** ‘Prognosis and longitudinal changes of physical activity in idiopathic pulmonary fibrosis’, BMC Pulmonary Medicine, 17(1). doi:10.1186/s12890-017-0444-0.
3. **Vainshelboim, B. et al. (2017)** ‘Lifestyle behaviors and clinical outcomes in idiopathic pulmonary fibrosis’, Respiration, 95(1), pp. 27–34. doi:10.1159/000481202.
